# Complete Genome Sequence of a Channel Catfish Epidemic Isolate, *Aeromonas hydrophila* Strain ML09-119

**DOI:** 10.1128/genomeA.00755-13

**Published:** 2013-09-19

**Authors:** Hasan C. Tekedar, Geoffrey C. Waldbieser, Attila Karsi, Mark R. Liles, Matt J. Griffin, Stefanie Vamenta, Tad Sonstegard, Mohammad Hossain, Steven G. Schroeder, Lester Khoo, Mark L. Lawrence

**Affiliations:** College of Veterinary Medicine, Mississippi State University, Mississippi State, Mississippi, USAa; Warmwater Aquaculture Research Unit, Agriculture Research Service, United States Department of Agriculture, Stoneville, Mississippi, USAb; Department of Biological Sciences, Auburn University, Auburn, Alabama, USAc; Bovine Functional Genomics Laboratory, Agricultural Research Service, United States Department of Agriculture, Beltsville, Maryland, USAd

## Abstract

*Aeromonas hydrophila* is a Gram-negative, rod-shaped, mesophilic bacterium that infects both aquatic poikilothermic animals and mammals, including humans. Here, we present the complete genome sequence of *Aeromonas hydrophila* strain ML09-119, which represents a clonal group of *A. hydrophila* isolates causing outbreaks of bacterial septicemia in channel catfish since 2009.

## GENOME ANNOUNCEMENT

*Aeromonas* species are Gram-negative facultative anaerobes that are ubiquitous in aquatic environments and cause infections in several host species, including humans, invertebrates, reptiles, and amphibians (1–5). In particular, many of the *Aeromonas* species are pathogenic to fish, causing septicemia in carp, tilapia, perch, salmon, catfish, and other species (6). In channel catfish aquaculture, *Aeromonas hydrophila* is historically considered an opportunistic pathogen. However, since 2009 a clonal group of *A. hydrophila* isolates have been causing large-scale disease outbreaks in Alabama and Mississippi (7). Strain ML09-119 is an isolate from a disease outbreak on a commercial catfish farm, and it is representative of this clonal group.

The genome sequence of *Aeromonas hydrophila* ML09-119 was completed using a combination of Illumina Genome Analyzer IIx next-generation sequencing (a total of 4,077,018 reads, with 104× coverage) (Illumina, Inc., San Diego, CA) (M. J. Hossain, G. C. Waldbieser, D. Sun, N. K. Capps, W. B. Hemstreet, K. Carlisle, M. J. Griffin, L. Khoo, A. E. Goodwin, T. S. Sonstegard, S. Schroeder, K. Hayden, J. C. Newton, J. S. Terhune, and M. R. Liles, submitted for publication) and the 454 GS-FLX titanium platform (a total of 96,601 reads with 308× coverage) (Roche Applied Science). Sequences from both platforms were assessed for errors and trimmed for quality using CLC workbench 5.0.1 (CLC Bio) and Sequencher 5.1 (Gene Codes Corporation). Assembly was performed by CLC workbench 5.0.1. Scaffolded gaps were closed by Sanger sequencing of PCR amplicons. Unscaffolded gaps were closed by sequencing single-primer PCR amplicons (8). rRNA operons and other repeat regions were amplified and sequenced to resolve misassemblies.

The final closed-circle version of the *A. hydrophila* ML09-119 genome sequence was submitted to the NCBI’s Prokaryotic Genomes Automatic Annotation Pipeline (PGAAP) (9) for annotation, followed by submission to GenBank. The total *A. hydrophila* genome comprises 5,024,500 bp with 60.8% GC content. It contains 4,577 predicted genes, of which 4,434 are protein-coding sequences. A total of 112 tRNAs and 10 rRNA operons were predicted by using tRNAscan-SE (10) and RNAmmer 1.2 (11), respectively.

The ML09-119 reads were assembled against the *A. hydrophila* ATCC 7966^T^ genome (NC_008570.1) in CLC workbench 5.0.1 to identify contiguous regions of the ML09-119 genome that are not present in the ATCC 7966^T^ genome. Functional analysis of predicted open reading frames (ORFs) in these unique contigs indicated that strain ML09-119 has a complete inositol utilization pathway that is not present in ATCC 7966^T^. More than 20 unique prophage-linked ORFs and several transposons were identified, and several putative virulence loci appear to be linked to prophage elements. Relative to strain ATCC 7966^T^, ML09-119 contains a unique 33-kb O polysaccharide biosynthesis gene cluster with 29 total predicted ORFs. Twenty-four of these do not have any similarity to ATCC 7966^T^ genes. Thus, it appears that ML09-119 has a different O antigen serotype than ATCC 7966^T^.

In summary, the *A. hydrophila* ML09-119 genome encodes putative proteins suggesting that it has unique biochemical and serological features relative to strain ATCC 7966^T^. Further analysis of these unique predicted ORFs (Hossain et al., submitted) showed that they are consistently present in other epidemic isolates and absent from *A. hydrophila* isolates that were not associated with epidemic outbreaks.

### Nucleotide sequence accession numbers.

The completed genome sequence of *A. hydrophila* ML09-119 was deposited in GenBank under the accession number CP005966, version CP005966.1.
